# Application of large language model combined with retrieval enhanced generation technology in digestive endoscopic nursing

**DOI:** 10.3389/fmed.2024.1500258

**Published:** 2024-11-06

**Authors:** Zhaoli Fu, Siyuan Fu, Yuan Huang, Wenfang He, Zhuodan Zhong, Yan Guo, Yanfeng Lin

**Affiliations:** ^1^Department of Gastroenterology, The Second Affiliated Hospital of Guanzhou University of Chinese Medicine, Guangzhou, China; ^2^The Fifth Affiliated Hospital of Guangzhou Medical University, Guangzhou, China

**Keywords:** large language model, retrieval enhanced generation technology, digestive endoscopic nursing, questionnaire survey scale, ChatGPT

## Abstract

**Background:**

Although large language models (LLMs) have demonstrated powerful capabilities in general domains, they may output information in the medical field that could be incorrect, incomplete, or fabricated. They are also unable to answer personalized questions related to departments or individual patient health. Retrieval-augmented generation technology (RAG) can introduce external knowledge bases and utilize the retrieved information to generate answers or text, thereby enhancing prediction accuracy.

**Method:**

We introduced internal departmental data and 17 commonly used gastroenterology guidelines as a knowledge base. Based on RAG, we developed the Endo-chat medical chat application, which can answer patient questions related to gastrointestinal endoscopy. We then included 200 patients undergoing gastrointestinal endoscopy, randomly divided into two groups of 100 each, for a questionnaire survey. A comparative evaluation was conducted between the traditional manual methods and Endo-chat.

**Results:**

Compared to ChatGPT, Endo-chat can accurately and professionally answer relevant questions after matching the knowledge base. In terms of response efficiency, completeness, and patient satisfaction, Endo-chat outperformed manual methods significantly. There was no statistical difference in response accuracy between the two. Patients showed a preference for AI services and expressed support for the introduction of AI. All participating nurses in the survey believed that introducing AI could reduce nursing workload.

**Conclusion:**

In clinical practice, Endo-chat can be used as a highly effective auxiliary tool for digestive endoscopic care.

## Introduction

With the rapid rise of OpenAI’s ChatGPT, large language models (LLMs) have attracted widespread attention in various fields. They have demonstrated significant capabilities in clinical information processing tasks, such as medical Q&A (1), data extraction (2), medical record summarization (3), content generation, and predictive modeling (4). However, commonly used large language models in the market, such as OpenAI’s ChatGPT, are trained using publicly available data and are not optimized for clinical use. This means that when prompted with clinical questions, publicly available LLMs may output incorrect, incomplete, or fabricated information and are unable to answer questions applicable to certain internal departmental data (5).

**Figure 1:**
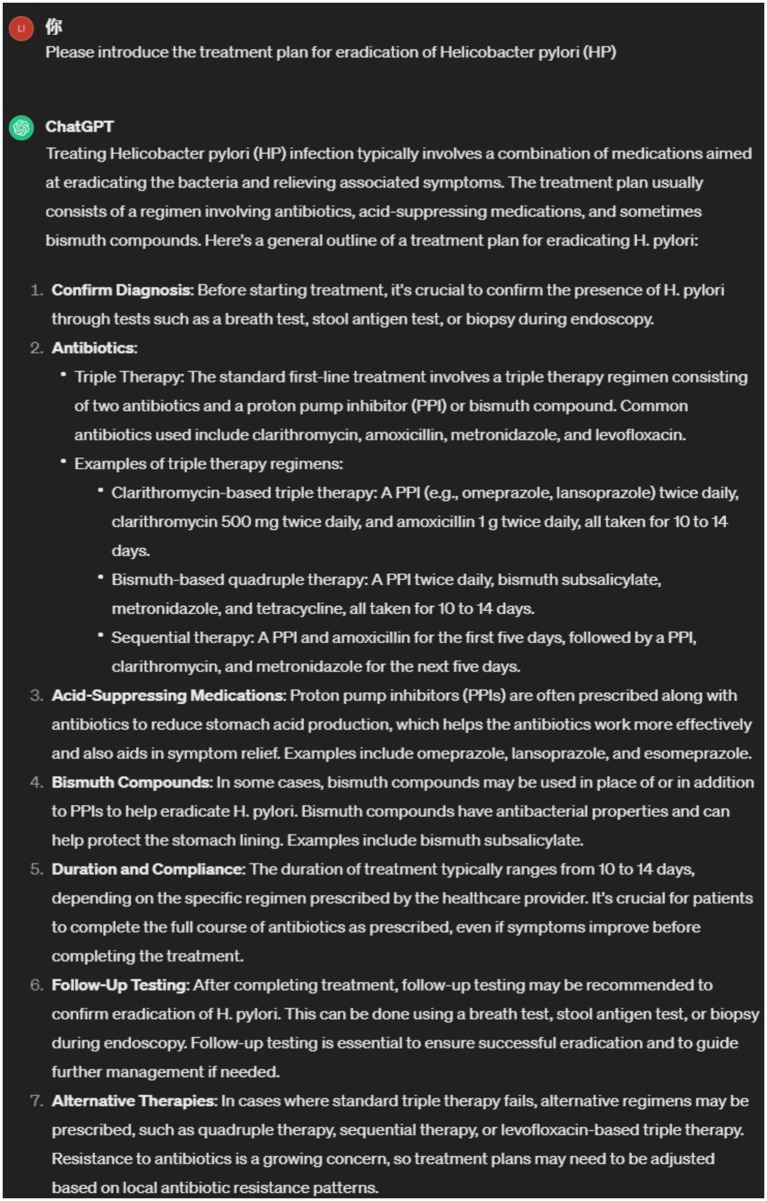
ChatGPT’s answer to treatment options to eradicate *H. pylori*.

**Figure 2:**
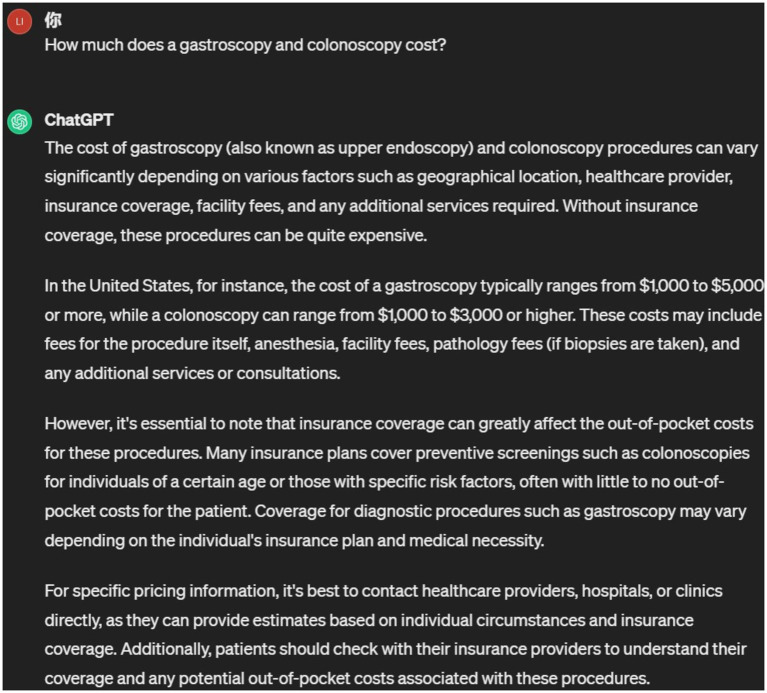
ChatGPT’s answer to gastroenteroscopy costs.

Despite these limitations, LLMs are believed to have enormous potential in biomedical and clinical applications. This is because modern medical practice is a highly complex task, with the volume of knowledge generated increasing annually. For example, it is estimated that in 2016, two papers were uploaded to PubMed every minute (6), which has surely increased over the past 7 years. The medical field continues to expand its clinical knowledge system and develop comprehensive practice guidelines, such as the 2022 Chinese guidelines for the treatment of *Helicobacter pylori* infection and the diagnosis and treatment of chronic gastritis. LLMs do not update in real-time or train on new medical guidelines, so they cannot answer more specialized questions in relevant disease areas. As shown in Figure 1, regarding the “eradication therapy for *H. pylori*,” ChatGPT’s response is vague and does not specifically address the specific scope and usage of dual, triple, or quadruple therapy regimens. Additionally, different hospitals and departments have different patient education content, such as where to pick up medications, how to schedule appointments with doctors, fee standards, etc., which LLMs cannot answer. As shown in Figure 2, ChatGPT cannot provide answers to this type of content. Currently, many studies have focused on how to use LLM to solve specific disease problems, but such research has found that the answers from LLM lack sufficient professionalism and cannot be compared to those from senior doctors (7–10). How to transform LLM to make its answers more domain-specific is currently a research hotspot.

There are three general methods and technologies to achieve the “specialization” of LLM: (1) Fine-tuning the original LLM model, which requires a large amount of computational resources that are generally unaffordable for hospital departments (11). (2) Using professional prompt words within LLM, which can only handle a small amount of data and requires users to constantly modify inputs (12–14). (3) Retrieval-augmented generation technology (RAG) (15–17).

In the era of large language models, RAG specifically refers to models retrieving relevant information from a vast document library when answering questions or generating text. Subsequently, this retrieved information is utilized to generate answers or text, thereby enhancing prediction accuracy. The RAG method allows developers to avoid retraining the entire large model for each specific task. Instead, they can provide additional information input to the model by attaching a knowledge base, thereby improving the accuracy of its responses. The RAG method is particularly suitable for tasks that require a large amount of knowledge. In this study, we adopted the RAG approach, collecting guidelines and internal departmental data related to gastroenterology and gastrointestinal endoscopy as a knowledge base. We combined Microsoft’s Azure OpenAI service as the large model to build an application for answering patient questions related to gastrointestinal endoscopy, known as Endo-chat.

## Method

### Ontology construction

Commonly used gastroenterology guidelines were downloaded from Wanfang and CNKI databases, and then integrated with internal departmental data to establish a knowledge base consisting of 17 documents (18–23, 27–36). The specific composition is shown in Table 1. Since the input knowledge base length for large models was limited, we had to divide the documents into smaller text blocks. Python 3.10 and llama index version 0.9.8 were used as the programming language and tool library (24). The Sentence Window Node Parser method was employed to split the documents, yielding a total of 752 knowledge base entries. After data preprocessing, each guideline entering the knowledge base has approximately 12,000 characters, totaling 194,582 characters. The internal departmental information has a total of 54,813 characters, making the grand total about 200,065 characters.

**Table 1:** Clinical guidelines and expert consensus related to digestive endoscopy included in the model.

No	Guide or document	Year
1	Internal department information	2024
2	Chinese National Clinical Practice Guideline on *H. pylori* Eradication Treatment	2022
3	Guidelines for diagnosis and treatment of chronic gastritis in China	2022
4	Clinical application guide of proprietary Chinese medicine in the treatment of ulcerative colitis	2022
5	Chinese Medical Association gastric cancer clinical guidelines	2022
6	Expert consensus on early diagnosis and treatment of esophageal cancer in China	2022
7	Consensus opinions on early screening and monitoring of high-risk groups of pancreatic cancer in China	2021
8	Chinese guidelines for screening and early diagnosis and treatment of gastric cancer	2022
9	Expert consensus on early esophageal cancer screening and endoscopic diagnosis and treatment in China	2014
10	Chinese guidelines for early colorectal cancer screening and endoscopic diagnosis and treatment	2014
11	Chinese population screening guidelines for liver cancer	2022
12	Clinical application guide of Magnetron capsule gastroscopy in China	2021
13	Primary liver cancer diagnosis and treatment guidelines	2022
14	Chinese guidelines for Diagnosis and treatment of acute pancreatitis	2021
15	Chinese guidelines for the application of endoscopic ultrasound-guided fine needle aspiration/biopsy	2021
16	Quick guide to intestinal preparation related to digestive endoscopic diagnosis and treatment in Chinese children	2020
17	Chinese guide for intestinal preparation related to digestive endoscopic treatment	2019

### RAG framework construction

**Figure 3:**
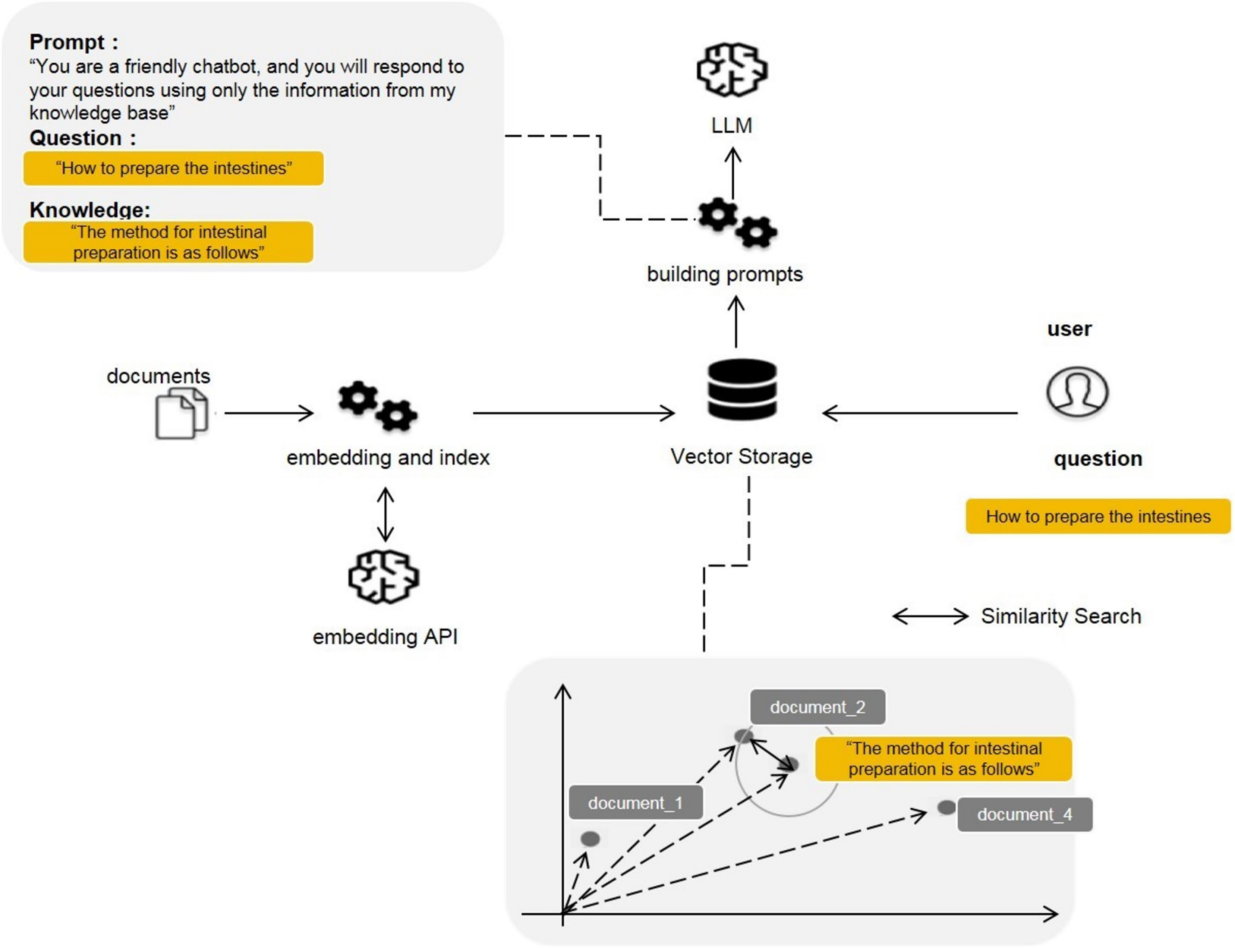
RAG frame for Endo-chat.

Integrating documents (Figure 3) was achieved through the utilization of the application programming interface (API) provided by Microsoft Azure OpenAI (15). In the preprocessing stage, the 752 knowledge base entries were embedded using the text-embedding-ada-002 model to convert the text into vector numerical representations. These vector values were further stored in a vector database, with Faiss database being used in this study. When patients input questions, such as “How to prepare for a colonoscopy,” they also undergo text embedding using the text-embedding-ada-002 model. The vector values of the patient’s input question are matched for similarity with the vector numerical values of the knowledge base entries in the vector database, filtering out the most relevant knowledge base entries. For example, a matched entry could be: “The method for colonoscopy preparation is as follows, dietary preparation…” We will retrieve 10 relevant knowledge articles and then let the large model determine which content to use as background knowledge to answer the user’s question. Finally, the patient’s question and the retrieved knowledge base entry are sent to the LLM, which answers the patient’s question based on prompt words and the knowledge base. The LLM selected for our Endo-chat is the gpt-3.5-turbo-16 k model.

### Interactive interface construction and effect evaluation

**Figure 4:**
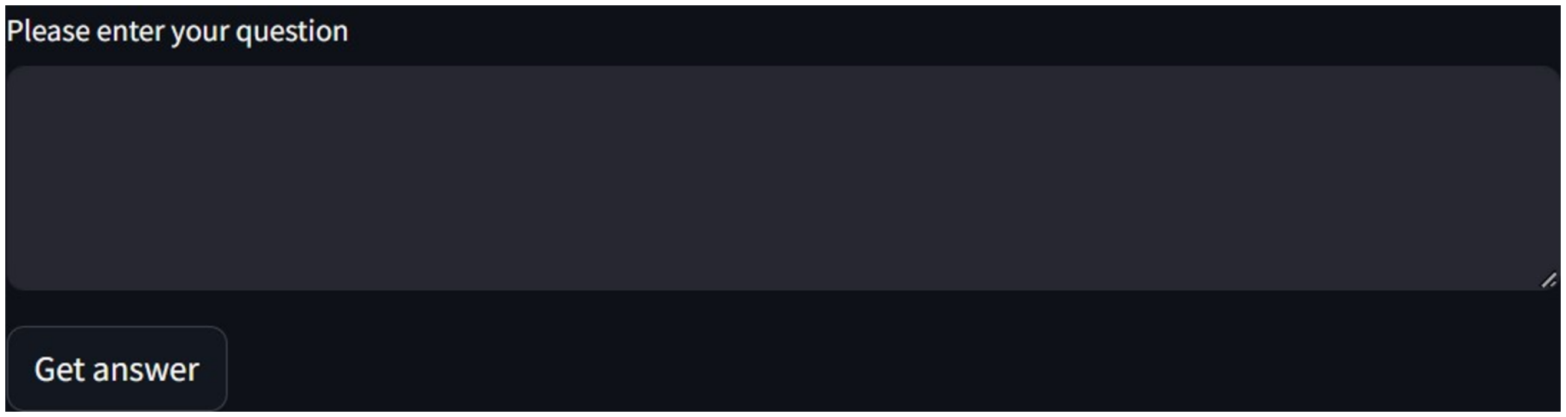
The simple chat interface of Endo-chat.

A user-friendly chat interface was designed using the streamlit tool library to enhance communication with patients (25). This interface, shown in Figure 4, enables patients to log in through a web link on their mobile phones or computers to inquire about any concerns they may have. To quantitatively evaluate the advantages of AI applications over traditional nurse manual responses to patient questions, we evaluate from the following aspects, as detailed in the supplement: (1) Efficiency Response time: comparing the average time required for AI robots and nurses to answer questions. (2) Accuracy Information accuracy: comparing the accuracy of the answers provided by the two methods through professional evaluation. Information completeness: evaluating whether the answers provided by the two methods comprehensively cover the patients’ inquiries. (3) Patient Satisfaction survey: assessing patient satisfaction with AI robots and manual services through a questionnaire survey. Preference test: asking patients which type of service they would prefer to use in the future. (4) Impact on nursing staff Workload: evaluating the changes in the workload of nursing staff after the implementation of AI robots. Job satisfaction: investigating the attitudes of nursing staff towards the introduction of AI robots and its impact on job satisfaction. 200 patients were randomly divided into two groups of 100 each to undergo gastrointestinal endoscopy examinations for a questionnaire survey.

## Results

To analyze the responses of Endo-chat and ChatGPT, we posed some typical gastroenterology questions to both. The comparison of their answers is presented in Table 2, where we focused on 5 specific questions. For questions with clear personalization, such as “How much does a gastrointestinal endoscopy cost,” ChatGPT is very cautious and provides answers that lack useful information. The Endo-chat, after matching with the department’s internal knowledge base, can provide very accurate answers. For questions with some level of personalization, such as “How should individuals preparing for a morning colonoscopy proceed,” ChatGPT’s responses were vague and lack practical significance, while Endo-chat provided specific and accurate answers. As for questions with higher levels of expertise, such as “Please introduce the treatment plan for eradicating *H. pylori*” and “How often should follow-up visits be conducted for a diagnosis of atrophic gastritis” Endo-chat combined the latest guidelines to provide precise answers, with response quality far superior to ChatGPT. To more thoroughly evaluate the performance of Endo-chat and ChatGPT in this specific field, we selected 200 questions and had them answered using both AI methods. Experienced doctors were invited to assess the responses from the two AIs, focusing on the accuracy and level of detail of the answers. Since the approach was akin to a competition, it was not suitable to use the chi-square test. Instead, we assigned “Win,” “Tie,” or “Lose” judgments for each comparison. “Win” indicated that Endo-chat provided a superior response, “Lose” meant that ChatGPT offered a better response, and “Tie” implied that there was no significant difference between the two answers. According to Table 3, in terms of accuracy, Endo-chat won in 70% of the 200 cases, and tied with ChatGPT in 26.5% of the cases. In terms of the level of detail in the responses, Endo-chat won 94.5% of the cases, and tied in 4%. In both accuracy and the level of detail in responses, Endo-chat significantly outperformed ChatGPT.

**Table 2:** Comparison of Endo-chat and ChatGPT responses to common digestive questions.

Questions	Endo-chat	ChatGPT	Source
How much does the gastroenteroscopy cost?	The cost of gastrointestinal endoscopy is as follows: The total cost of painless gastroscopy is about 900 yuan, of which, apart from the medication used to remove gastric mucus during the gastroscopy (70 yuan), the remaining costs are reimbursable by medical insurance. The total cost of painless colonoscopy is about 1,000 yuan, reimbursable by medical insurance. The total cost of painless gastroscopy + colonoscopy is about 1,600 yuan, reimbursable by medical insurance. If colonic polyps requiring surgical treatment are found during the examination, and the patient is insured under the Guangzhou Employee Medical Insurance, the single disease settlement method can be used, and the patient only needs to pay 20% of the total cost.	I cannot provide real-time medical cost information because these costs may vary depending on the region, medical institution, and specific examination items. Generally, medical expenses may differ based on factors such as the patient’s geographical location, the level of the hospital, and the experience of the doctor. The best way is to directly contact hospitals or medical institutions near you to inquire about the specific costs of gastrointestinal endoscopy examinations. Usually, the hospital’s customer service or finance department will provide such information.	Department internal information – Fee description
How should individuals preparing for a morning colonoscopy proceed with bowel preparation?	For individuals undergoing a morning colonoscopy, the bowel preparation method is as follows: Dietary adjustments: Avoid consuming foods with many seeds, such as kiwi, dragon fruit, etc., two days before the examination to prevent affecting the colonoscopy results. Adopt a semi-liquid diet the day before the examination, avoiding meat, vegetables, fruits, but consuming easily digestible foods like fish, soft-boiled eggs, tofu to reduce the burden on the intestines. Dinner time and fasting water: It is recommended to finish dinner before 20:00 the day before, start fasting after 20:00, but water intake is allowed. Stop drinking water from 6:00 am on the day of the examination to ensure adequate bowel preparation. Use of laxatives: Start taking the first dose of laxative at 21:30: Mix one packet of polyethylene glycol electrolyte powder with 1,000 mL of water and drink it within an hour, then continue drinking water. During the laxative intake, be active, massage the abdomen to promote bowel movements; the main purpose is to move food from the gastrointestinal tract to the small and large intestines. Take the second dose of laxative at 4:00 am: Mix two packets of polyethylene glycol electrolyte powder with 2000 mL of water and drink it within 1.5 h. Then, drink 100 mL of warm water mixed with a bottle of simethicone, followed by 500–1000 mL of water. Stop drinking water from 6:00 am on the day of the examination. Be active and massage the abdomen during laxative intake to promote bowel movements. Typically, bowel movements start about an hour after taking the laxative, with the peak of diarrhea occurring around 1 h later. Precautions: It is better to drink the medication quickly but be cautious to avoid vomiting because the laxative is a volume-based one, and a large amount of fast-acting medication can enhance intestinal flushing efficiency. Stool should be clear water-like (or light tea-like) to meet the examination standards. If there are still residues or the stool is cloudy in the last bowel movement, please arrive at the endoscopy center before 08:30, seek help from medical staff, and take remedial measures. By following the above bowel preparation steps, the accuracy and smooth progress of the colonoscopy examination can be ensured. Please carefully follow the preparation steps according to the medical advice to obtain the best examination results.	Preparation before a colonoscopy is crucial to ensure the accuracy and effectiveness of the examination. Here are the general preparation steps before a colonoscopy: Clear liquid diet: Typically, one or two days before the colonoscopy, the doctor will recommend a clear liquid diet, which involves consuming only transparent liquids such as clear soup, fruit juice, jelly, tea, etc. Avoid foods high in fiber, fats, or residues. Taking laxatives or enemas: The doctor may require the patient to take laxatives or undergo an enema to help clear the intestines for a smoother examination. The type and usage of laxatives will depend on the doctor’s advice. Follow medical instructions: Act according to the specific instructions provided by the doctor or medical institution, including when to start the clear liquid diet, when to take laxatives or undergo an enema, etc. Stop consuming solid foods: Typically, for a certain period before the colonoscopy, patients need to stop eating solid foods to ensure thorough emptying of the intestines. Ensure transportation and accompaniment arrangements: Since a colonoscopy involves inserting a camera through the anus into the rectum and colon, which may cause some discomfort and be affected by anesthesia, patients need to arrange transportation and preferably have someone accompany them. Before undergoing a colonoscopy, it is essential to follow the specific instructions provided by the doctor or medical institution to ensure a smooth and accurate examination. Additionally, if there are any questions or concerns, be sure to communicate with the doctor promptly.	Internal data of the department – Instructions for bowel preparation
Please introduce the treatment plan to eradicate *H. pylori*	The treatment plan for eradicating *H. pylori* usually involves different combinations of drugs and courses. The following are various schemes detailed based on the text you provided: High-dose dual therapy: Use lansoprazole or rabeprazole (twice the standard dose, twice daily, or standard dose, four times daily) in combination with amoxicillin (at least 3.0 grams daily, such as 1.0 gram per dose, three times daily or 0.75 grams per dose, four times daily), using a proton pump inhibitor (PPI) such as esomeprazole. Triple therapy: Combination treatment with a PPI and two antibacterial drugs. Bismuth quadruple therapy: Combination treatment with a PPI and bismuth, along with two antibacterial drugs. Non-bismuth quadruple therapy: Combination treatment with a PPI and three antibacterial drugs (amoxicillin, clarithromycin, and metronidazole). Concomitant therapy: Treatment with a PPI combined with amoxicillin, clarithromycin, and metronidazole for 10–14 days. Sequential therapy: Phase 1: Treatment with a PPI combined with amoxicillin for 5–7 days. Phase 2: After the first phase, treatment with a PPI combined with clarithromycin and metronidazole for another 5–7 days. Hybrid therapy: Mixed application of sequential and concomitant therapy. Phase 1: Treatment with a PPI combined with amoxicillin for 5–7 days. Phase 2: After the first phase, treatment with a PPI combined with amoxicillin, clarithromycin, and metronidazole for another 5–7 days. When selecting the appropriate treatment plan, doctors typically consider the individual circumstances of the patient, past medication history, and potential drug resistance. Completing the entire treatment course is crucial to ensure the eradication of *H. pylori* and reduce the risk of recurrence. Additionally, close monitoring of the patient’s symptoms, *H. pylori* infection status, and possible adverse drug reactions is necessary during and after treatment.	The treatment regimen for *H. pylori* infection typically involves a combination of antibiotics and other medications. Below is a general treatment plan: Antibiotic therapy: Commonly used antibiotics include clarithromycin, amoxicillin, and metronidazole. Doctors may choose appropriate antibiotics based on local resistance patterns and the patient’s allergy history. Proton pump inhibitors (PPIs): PPI medications such as omeprazole, lansoprazole, which reduce gastric acid secretion, help enhance the effectiveness of antibiotics. They are usually used in conjunction with antibiotics. Other medications: Additional medications may be added, such as gastric mucosal protectants, to reduce gastric mucosal damage. Bismuth and gastric mucosal protectants like bismuth citrate are also commonly used in combination with antibiotics. Duration and dosage of treatment: The specific treatment plan will vary based on the patient’s individual circumstances and the doctor’s recommendations. Generally, treatment lasts for about one to two weeks. After treatment completion, testing for *H. pylori* infection is typically conducted to confirm successful bacterial eradication. This may include breath tests, serological tests, or other diagnostic methods. Following treatment completion, some patients may require further follow-up to ensure the infection does not recur. Please note that treatment plans may vary based on individual differences, the severity of the condition, and resistance patterns. Therefore, it is advisable to follow the doctor’s advice and adhere to their prescribed treatment.	Chinese guidelines for the treatment of *H. pylori* infection
What treatment plan should be selected for the primary eradication of *H. pylori* infection	For the eradication treatment of initial *H. pylori* (Hp) infection, the recommended regimen is bismuth quadruple therapy, which includes proton pump inhibitors and bismuth, selecting appropriate antibiotic combinations based on the patient’s condition. Here are detailed treatment recommendations: Treatment plan: Regimen selection: Bismuth quadruple therapy (including proton pump inhibitors and bismuth). Duration: 14 days. Drug combination: Choose the appropriate antibiotic combination based on the patient’s condition, recommended antibiotic combinations are listed in Table 3. Implementation recommendations: Communication with the patient: Before using bismuth quadruple therapy containing tetracycline and metronidazole, thorough communication with the patient should be conducted to inform them of possible adverse reactions. Consider resistance patterns: For patients in areas with high resistance to clarithromycin, levofloxacin, and metronidazole, or those with a history of prior antibiotic use, the possibility of difficult eradication should be considered, and bismuth quadruple therapy containing furazolidone may be selectively chosen. Recommended drug combinations: Recommended combination 1: Amoxicillin 1.0 g, twice daily Furazolidone 100 mg, twice daily Proton pump inhibitor (such as omeprazole, etc.) Bismuth (such as colloidal bismuth) Recommended combination 2 (considering resistance patterns): Amoxicillin 1.0 g, twice daily Furazolidone 100 mg, twice daily Tetracycline 500 mg, 3–4 times daily Proton pump inhibitor Bismuth Eradication rate comparison: Bismuth quadruple therapy eradication rate: Average of 81.3%. Triple therapy eradication rate: Average of 71.3%. Benefits outweigh risks: Although bismuth quadruple therapy may increase certain adverse reactions, overall, it has a higher eradication rate, and the adverse reactions are mostly non-severe types, thus the benefits outweigh the risks. In conclusion, for the eradication treatment of initial *H. pylori* infection, bismuth quadruple therapy is the recommended first-line regimen. However, when selecting specific drug combinations, the patient’s resistance patterns and individual characteristics should be considered to maximize treatment effectiveness and tolerability.	The initial eradication treatment for *H. pylori* infection typically involves a combination of antibiotic therapy to reduce bacterial resistance and improve treatment success rates. One of the commonly used treatment regimens is as follows: Triple therapy: Triple therapy is one of the most commonly used regimens for eradicating *H. pylori* infection, consisting of a proton pump inhibitor (PPI) in combination with two antibiotics. Common triple therapy regimens include: Proton Pump Inhibitor (PPI) + Clarithromycin + Amoxicillin Proton Pump Inhibitor (PPI) + Clarithromycin + Metronidazole Quadruple therapy: Quadruple therapy adds an additional antibiotic compared to triple therapy, typically comprising a PPI and a combination of three antibiotics. Common quadruple therapy regimens include: Proton Pump Inhibitor (PPI) + Clarithromycin + Amoxicillin + Metronidazole Quintuple therapy: Quintuple therapy is a newer treatment approach, including a PPI, two antibiotics, and a gastric mucosal protectant. Common quintuple therapy regimens include: Proton Pump Inhibitor (PPI) + Clarithromycin + Amoxicillin + Bismuth subsalicylate The selection of treatment regimens should be based on the patient’s specific condition, local antibiotic resistance patterns, and past treatment history. Additionally, close monitoring of the patient’s symptoms and resistance patterns should be conducted during treatment, with adjustments made as necessary. Ultimately, eradicating *H. pylori* infection requires strict adherence to the doctor’s instructions throughout the entire treatment process.	Chinese guidelines for the treatment of *H. pylori* infection
How often should atrophic gastritis be followed up	Based on the provided information, the follow-up frequency recommendations for atrophic gastritis are as follows: For mild atrophic gastritis limited to the gastric antrum, regular endoscopic follow-up may not be necessary. For atrophic gastritis involving the gastric body, it is recommended to undergo endoscopy every 1 to 3 years. When accompanied by other gastric cancer risk factors such as a family history of gastric cancer, incomplete intestinal metaplasia, extensive atrophy and metaplasia, persistent *H. pylori* infection, closer endoscopic follow-up is required. For patients with severe atrophic gastritis and a family history of gastric cancer, annual endoscopic follow-up is recommended. For patients with histological results of dysplasia but no clear lesions found during conventional white light endoscopy, immediate high-definition endoscopy is recommended, with suspicious lesions biopsied. For patients with biopsy results showing high-grade intraepithelial neoplasia or high-grade dysplasia, immediate re-examination with high-definition endoscopy and consideration of endoscopic treatment or even surgical intervention is advised. For patients with biopsy results showing low-grade intraepithelial neoplasia or low-grade dysplasia, follow-up with high-definition endoscopy is recommended within 6 months to 1 year. American scholars suggest that even for moderate to severe atrophic gastritis, endoscopic monitoring every 3 years is sufficient, which may be related to the high cost of endoscopy and the low incidence of gastric cancer. Risk factors related to gastric cancer include *H. pylori* infection, family history of gastric cancer, pernicious anemia, high-risk areas for gastric cancer, ≥15 years post-subtotal gastrectomy, age ≥ 45 years, male gender, history of high salt intake, and smoking history, all of which should be considered. For lymphocytic gastritis, regular endoscopic follow-up is recommended to monitor the development of lymphoma. To improve the positivity rate of biopsies and the accuracy of monitoring follow-up, targeted optical biopsies or gastric mucosal standardized live tissue examinations may be considered when conditions permit. In conclusion, the follow-up frequency should be determined based on the patient’s specific condition and pathological results, including the extent of the lesion, existing risk factors, and the availability of medical resources. Therefore, it is recommended to further discuss with medical experts and develop the most appropriate follow-up plan based on individual circumstances.	After being diagnosed with atrophic gastritis, the frequency of follow-up should be determined by the doctor based on the specific condition of the patient. Generally, the frequency of follow-up may vary due to individual differences, the severity of the condition, and the response to the treatment plan. Typically, doctors may recommend intensive follow-up after the initial diagnosis to ensure effective control of the condition and assess the treatment outcomes. Once the condition stabilizes, the frequency of follow-up may gradually decrease, but regular examinations are still necessary to monitor changes in the condition and adjust the treatment plan. It is advisable to communicate with the attending physician to understand the best follow-up plan tailored to your individual situation. Additionally, following the treatment and follow-up recommendations of the doctor is crucial to ensure effective management of your health.	Guidelines for diagnosis and treatment of chronic gastritis in China

**Table 3:** Evaluation of the quality of responses from two AI methods.

	Win	Tie	Lose
Accuracy	140 (70%)	53 (26.5%)	7 (3.5%)
Level of detail	189 (94.5%)	8 (4%)	3 (1.5%)

A questionnaire evaluation was performed on two approaches, AI and manual, as outlined in Table 4. There were no statistically significant differences in age and gender between the two groups of patients. In terms of response efficiency, completeness, and patient satisfaction, AI was significantly superior to the manual method. In terms of response accuracy, the manual group, which was considered to have very accurate answers, accounted for 91%, while the AI group accounted for 82%. There was no significant difference between the two groups (*p* = 0.085). For each participant, we provided a detailed introduction to both the AI and manual methods, so participants had a certain degree of understanding of the other method. Regarding the question “which services do you prefer to use in the future?” we are investigating whether participants are dissatisfied with the method they are currently using, rather than which method they prefer. Among the participants in the manual group, 45% were willing to try the method of the AI group. However, only 20% of the participants in the AI group were willing to switch to the manual method. There was a significant statistical difference between the two (*p* < 0.001). Both groups of participants held a supportive attitude towards the introduction of AI, and there was no statistical difference between the two groups (*p* = 0.2485). All nurses participating in the questionnaire believed that introducing AI could reduce nursing workload. In open-ended questions, the most common suggestion from patients was to introduce a combination of AI and manual methods, where manual intervention could be provided for questions that AI could not answer or had doubts about. It is evident that the use of AI methods significantly helps improve nursing efficiency and enhance patient satisfaction.

**Table 4:** Evaluation of Endo-chat based method compared with manual method.

	AI group	Manual group	χ^2^	*p*
**Age**			1.0898	0.7795
18–30	18	15		
31–45	23	28		
46–60	34	30		
61–~	25	27		
**Gender**			1.302	0.2538
Male	48	39		
Female	52	61		
**After you ask the AI/nurse a question, how long will you receive an answer?**			106.6876	<0.001
Immediate	91	18		
Less than 5 min	3	17		
5–30 min	0	23		
More than 30 min	7	42		
**How accurate do you think the answers are?**			6.6111	0.085
Very precise	82	91		
Precise	9	5		
With some accuracy	4	4		
Inaccuracy	5	0		
**Does the answer fully cover your question?**			20.4669	<0.05
Complete cover	88	71		
Basic overlay	10	6		
Only partially covered	1	13		
Barely cover	1	10		
**How satisfied are you with the service overall?**			11.083	<0.05
Very satisfied	54	41		
Satisfied	32	25		
Generally satisfied	10	26		
Dissatisfied	4	8		
**Which services do you prefer to use in the future? (If it is an AI group, then this method is AI)**			19.1425	<0.001
The method of this experiment	56	30		
Another method of this experiment	20	45		
Indifferent	10	15		
Uncertainty	14	10		
**What is your attitude towards the introduction of AI robots?**			2.7848	0.2485
Supportive	76	72		
Indifferent	20	18		
Disagree	4	10		

## Discussion

In the clinical work of digestive endoscopy, nurses often need to answer a large number of questions regarding preoperative, postoperative, diet, follow-up, etc., which consumes a significant amount of working time. For routine examination patients, these questions can be answered using standardized language or procedures, for example, the question “How should I prepare for a gastrointestinal endoscopy?” can be answered based on the department’s internal processes. However, for patients with different diagnoses, a certain level of medical expertise is required. For example, the question “How long should I wait for a follow-up colonoscopy?” would need personalized advice based on clinical guidelines. Research has shown that using large-scale language models (such as ChatGPT) for medical Q&A in clinical practice can improve the efficiency of medical and nursing work. However, there are two major obstacles to the use of large language models in the medical field: one is that these models may generate “fictional” or seemingly credible but incorrect answers, which is an inevitable issue with large models; the other is the inability to answer personalized questions related to specific departments, personal health information, etc. Due to the low tolerance for errors in medical Q&A, these two issues need to be addressed urgently. Therefore, we combined RAG with LLM to create the Endo-chat Q&A application, which not only alleviates the above two issues to a large extent but also significantly improves nursing work in clinical practice.

In our experiment, the specific practice method involved nurses replying to patient questions in a WeChat group, which consumed a significant amount of nursing effort. Additionally, nurses were unable to provide detailed answers to multiple questions from multiple patients simultaneously. In contrast, the advantages of the AI method are evident. Endo-chat is a 24/7 online robot that can provide immediate feedback to patient questions. Due to the use of the RAG technology framework, its response accuracy is comparable to that of humans, and it can provide more detailed and comprehensive answers than manual responses.

This study also has certain limitations. (1) The RAG framework may generate incorrect answers due to inaccurate retrieval of knowledge articles, although this possibility is small. As shown in Figure 3, the construction of Endo-chat can be divided into three parts: ① Creating a knowledge base using document segmentation; ② Creating a vector database; ③ Matching and retrieving questions with the knowledge base. The retrieved knowledge articles and questions are entered into the LLM for answer generation. If the retrieval of knowledge articles is inaccurate and LLM answers based on incorrect knowledge, it may lead to an incorrect answer. This is why there were 5 cases of inaccurate AI responses in the question “How accurate do you think the answers are?” in Table 4. To minimize the occurrence of inaccurate recalls, we drew inspiration from the Self-RAG (26) approach. We first recalled 10 texts based on the similarity between the question and the knowledge passage embeddings. We then allowed the LLM to analyze in parallel whether these 10 texts were relevant to the question, ultimately only incorporating the relevant knowledge passages as the background for Endo-chat’s responses. For example, if the user input is “Hello, who are you?” Endo-chat will not adopt any knowledge passages. Conversely, if all 10 passages recalled are closely related to the question, Endo-chat will adopt all of them. The question of how many texts need to be recalled to obtain the best response is an engineering problem, which depends on the total number of knowledge passages, the required response speed, and the context length limit of the LLM. Building a higher-quality knowledge base and achieving more precise recalls is an area worth exploring in the future. (2) LLM may also fail to follow instructions, even when the correct knowledge articles are input, LLM may not answer according to the knowledge articles. This may be due to the preference selection during LLM training. (3) This study only included 17 documents and cannot cover all content in the field of gastroenterology. LLM can only accurately answer the content contained in these documents. If a patient asks about content outside the documents, such as “What should I do for chronic diarrhea” LLM can only answer based on its own capabilities, resulting in a general response. (4) Due to the use of an external API, Endo-chat may not be able to respond when there are many concurrent requests. This can explain the question “How long does it take to receive an answer after asking the AI robot/nurse?” where in some cases, AI cannot respond promptly.

In conclusion, combining RAG technology and using LLM for medical vertical domain Q&A is a meaningful clinical practice. Our next step could involve optimizing the RAG framework, expanding the content of the knowledge base, and making Endo-chat responses more accurate and applicable.

## Data Availability

The raw data supporting the conclusions of this article will be made available by the authors, without undue reservation.

## Appendix: Questionnaire survey scale

### Section 1: Basic information

1. Your age:

- 18–30
- 31–45
- 46–60
- 61 and above

2. Your gender:

- Male
- Female
- Other
- Prefer not to say

3. Your colonoscopy date:

- ____ (Please fill in the date)

### Section 2: Service efficiency

1. How long did it take for you to receive a response after asking a question to the AI robot/nurse?

- Immediately
- Less than 5 minutes
- 5–30 minutes
- More than 30 minutes

### Section 3: Accuracy and completeness

1. How accurate do you think the answers were?

- Very accurate
- Accurate
- Somewhat accurate
- Inaccurate

2. Did the answer fully cover your questions?

- Completely covered
- Mostly covered
- Partially covered
- Hardly covered

### Section 4: Patient satisfaction

1. How satisfied are you with the overall service?

- Very satisfied
- Satisfied
- Neutral
- Dissatisfied

2. Which service would you prefer to use in the future?

- AI robot
- Nurse manual
- No preference
- Undecided

### Section 5: Impact on nursing staff (if applicable)

1. If you are a nursing staff, how has your workload changed after the introduction of the AI robot?

- Significantly reduced
- Slightly reduced
- No change
- Slightly increased
- Significantly increased

2. What is your attitude towards the introduction of the AI robot?

- Strongly support
- Support
- Neutral
- Oppose
- Strongly oppose

### Section 6: Open-ended questions

1. What suggestions or comments do you have for the AI robot service?

- _____________________________________________

2. What suggestions or comments do you have for the nurse manual service?

- ______________________________________________
